# Eliminating Digestive Irregularities Caused by Late Effects: A Pilot Study of an Innovative Culinary Nutrition Intervention for Reducing Gastrointestinal Toxicity in Gynecologic Cancer Patients Who Have Undergone Pelvic Radiotherapy

**DOI:** 10.3390/nu16234227

**Published:** 2024-12-06

**Authors:** Cheryl Pritlove, Geremy Capone, Mathankki Ramasamy, Lisa Avery, Daniela Fierini, Sarah E. Ferguson, Kathy Han, Jennifer M. Jones

**Affiliations:** 1 Li Ka Shing Knowledge Institute, Unity Health Toronto, Toronto, ON M5C 2T2, Canada; cheryl.pritlove@unityhealth.to (C.P.); mathankki.ramasamy@mail.utoronto.ca (M.R.); 2 Dalla Lana School of Public Health, University of Toronto, Toronto, ON M5S 1A, Canada; 3 Cancer Rehabilitation and Survivorship Program, Princess Margaret Cancer Centre, Toronto, ON M5G 2C4, Canada; geremy.capone@uhn.ca (G.C.); daniela.fierini2@uhn.ca (D.F.); 4 Department of Biostatistics, Princess Margaret Cancer Centre, Toronto, ON M5G 2C4, Canada; lisa.avery@uhn.ca; 5 Division of Gynecologic Oncology, University Health Network/Sinai Health Systems, Toronto, ON M5G 1X5, Canada; sarah.ferguson@uhn.ca; 6 Department of Obstetrics and Gynecology, University of Toronto, Toronto, ON M5S 1A, Canada; 7 Radiation Medicine Program, Princess Margaret Cancer Centre, University Health Network, Toronto, ON M5G 2C4, Canada; kathy.han@uhn.ca; 8 Department of Radiation Oncology, University of Toronto, Toronto, ON M5S 1A, Canada; 9 Department of Psychiatry, Faculty of Medicine, University of Toronto, Toronto, ON M5S 1A, Canada

**Keywords:** gynecologic cancer, cancer survivorship, gastrointestinal toxicity, nutrition, culinary

## Abstract

Background/Objectives: Pelvic radiotherapy (RT) improves survival in gynecologic cancer patients but often results in gastrointestinal (GI) toxicity, affecting quality of life. Standard nutrition guidance lacks specificity for these survivors, complicating dietary choices. To address this gap, the EDIBLE intervention was developed to offer structured dietary self-management skills to alleviate RT-induced GI toxicity. Methods: We conducted a single-arm mixed-methods pilot of the EDIBLE intervention among post-treatment gynecologic cancer survivors to assess its feasibility, acceptability, and preliminary effects on GI symptoms, knowledge, and self-efficacy, with measures at baseline (T1), post-intervention (T2), and after 3 months (T3). Results: Qualitative interviews supported strong perceptions of intervention feasibility; however, the recruitment (32%) and retention (72%) rates were modest, indicating that alternate formats for program delivery may be needed to make it more accessible. The acceptability of the EDIBLE intervention garnered especially high ratings on measures of satisfaction and utility, with program improvements largely rallying around a desire for increased in-class sessions and program expansion. Statistically significant improvements were observed at the three-month mark (T3), such as enhanced confidence in culinary practices, increased knowledge and skills with regard to managing GI side effects, and improvements in bowel and GI symptoms. Conclusions: The results suggest EDIBLE is acceptable, improving GI symptoms and self-efficacy; however, moderate recruitment rates indicate refinement is needed. A randomized control trial and cost-effectiveness analysis is needed to confirm effectiveness and scalability.

## 1. Introduction

Pelvic radiotherapy (RT) plays a key role in the curative treatment of patients with gynecologic cancers [1]. RT techniques have improved significantly over the past 30 years, benefiting patients who undergo treatment for pelvic malignancies [2,3,4] and contributing to a growing population of gynecologic cancer survivors [5,6,7,8,9]. Despite significant advancement in RT techniques and resultant survival rates, radiation-induced gastrointestinal (GI) toxicity remains common in this population [2,10,11,12]. Up to 80% of gynecologic cancer survivors treated with curative doses of abdominal or pelvic radiation experience significant acute GI toxicity (occurring within 90 days), including fecal leakage, uncontrolled fecal emptying, flatulence, bloating and rectal bleeding [12,13,14,15], which can significantly impact their quality of life (QoL) [12,13,16,17,18,19,20]. While these symptoms may resolve following the end of RT, up to 50% of patients with GI toxicity experience these symptoms for >90 days [12,13,14], with some reporting symptoms lasting up to a decade post-treatment [17,21,22]. The effective management of GI toxicity in cancer survivors is integral to optimizing nutritional intake and maintaining long-term digestive health, as well as promoting improved well-being and a higher QoL [23,24,25].

Clinical guidelines recommend suitable and efficient nutrition therapy to help address nutritional challenges experienced by cancer patients’ post-treatment [26]. However, general nutrition advice provided for cancer survivors often does not address the specific dietary challenges and needs of individuals coping with GI side effects and digestive complications [27]. While specific guidelines for the nutrition management of GI side effects in gynecologic cancer survivors do not currently exist [28], dietary adjustments have been proposed as possible mitigators of GI toxicity from RT; such adjustments include following a low-residue/modified fiber diet, which limits insoluble fiber, fat, lactose, spicy foods, sugar alcohols and caffeine [28,29,30,31,32,33].

Due to the high degree of variability in the pathophysiology of GI symptoms, trial and error is a necessary part of self-management after treatment [31,34]. However, many gynecologic cancer survivors are afraid to try foods that may exacerbate their symptoms, even if smaller portions may be well tolerated [33]. This can lead to reduced variety in the diet, which may compromise the nutritional status of the cancer survivor, who may already have compromised nutrient absorption and food intake [35]. Many gynecologic cancer survivors express frustration around trying to follow general guidelines on healthy eating for cancer survivors [36], as many of the promoted foods seem to worsen GI side effects [33]. Tailored nutrition education can be helpful in encouraging dietary variety and experimentation with foods and serving sizes that will be less likely to worsen side effects [33,35]. Providing structured self-management support may thus be a helpful first step to improve the QoL of gynecologic cancer survivors who suffer from post-radiation GI toxicity.

Despite the increasing number of gynecological cancer survivors, the high burden of RT-related GI side effects [37], and a growing body of data that identify the role that diet can play in worsening or improving symptoms, dietary self-management support programs addressing radiation treatment-induced GI toxicity have yet to be developed and tested. Thus, we developed a structured dietary self-management skills intervention entitled **E**liminating **D**igestive **I**rregularities caused **B**y **L**ate **E**ffects (EDIBLE) and piloted it with gynecologic cancer survivors post pelvic RT and reported symptoms of late GI toxicity.

## 2. Methods and Materials

We conducted a single-arm mixed-methods pilot study of a supported self-management dietary intervention (EDIBLE) among post-treatment gynecologic cancer survivors at the Princess Margaret Cancer Centre in Toronto, Canada. The research aims were to (1) assess the feasibility of the experimental methods and intervention; (2) assess the acceptability and perceived helpfulness of the intervention; and (3) obtain a preliminary estimate of the effect of the intervention on bowel and GI symptoms, knowledge, and self-efficacy. Outcome measures were administered at baseline (T1), immediately post-intervention (T2), and again 3 months later (T3). Qualitative interviews were conducted at T3.

### 2.1. Description of Intervention

**Figure 1:**
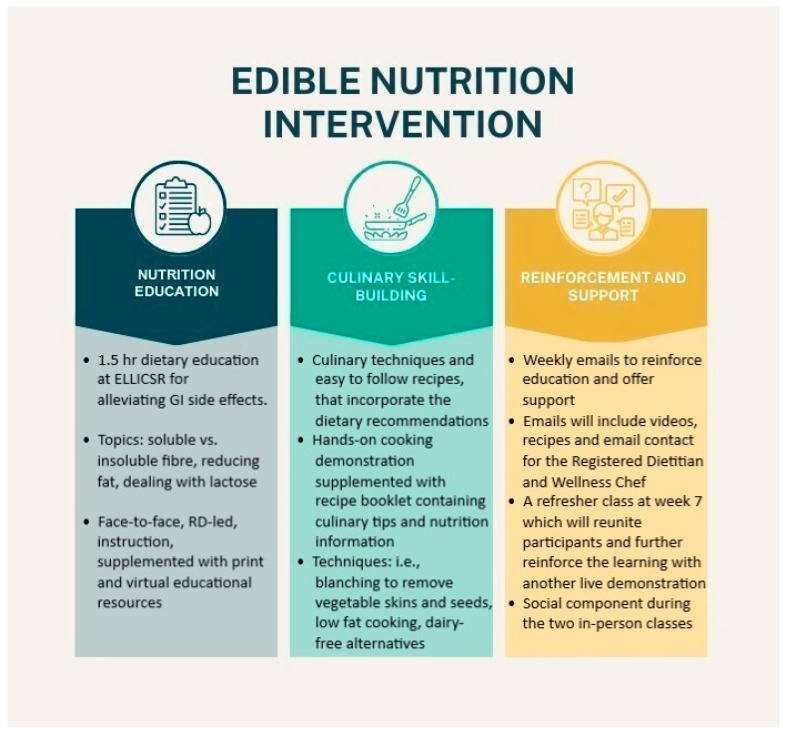
Three pillars of the EDIBLE program.

The development of EDIBLE was guided by several well-established behavior change theories [38,39] and related techniques known to be linked to sustained positive outcomes for both cooking skills and dietary habits [40], including demonstration of skills; identification of barriers/problem solving with regard to implementation; and prompting practice [41]. The program was composed of three pillars: nutrition education, culinary skill-building, and reinforcement and support (see Figure 1).

The nutrition education component was designed in consultation with evidence recommending a diet limiting foods that may worsen GI side effects, such as insoluble fiber, fat, lactose, spicy foods, sugar alcohols and caffeine [31,33]. Limiting insoluble fiber, which is found in whole grains, beans, fruits, vegetables, nuts and seeds, seems to be the most effective dietary strategy for improving GI symptoms in gynecologic cancer survivors who have received chronic radiation enteritis [33,42,43,44,45]. In contrast, increasing soluble fiber intake is recommended for managing diarrhea and fecal incontinence, as it forms a gel-like substance with the excess liquid in the digestive tract, thereby slowing the intestinal transit time [46,47]. Studies have also demonstrated that reducing fat intake to less than 40 g per day can decrease the severity of diarrhea [48,49], and that a diet low in lactose, fat, and fiber may further alleviate radiation enteritis symptoms [50]. Informed by this evidence, the EDIBLE intervention included nutrition education and recipes that promoted strategies to minimize dietary intake of foods that are higher in lactose, insoluble fiber, and fat.

The key learning objectives for participants within the intervention were as follows: (1) understand how food choices and food preparation can affect the occurrence and severity of GI irregularities and (2) apply culinary techniques that reduce the occurrence and severity of GI irregularities.

**Figure 2:**
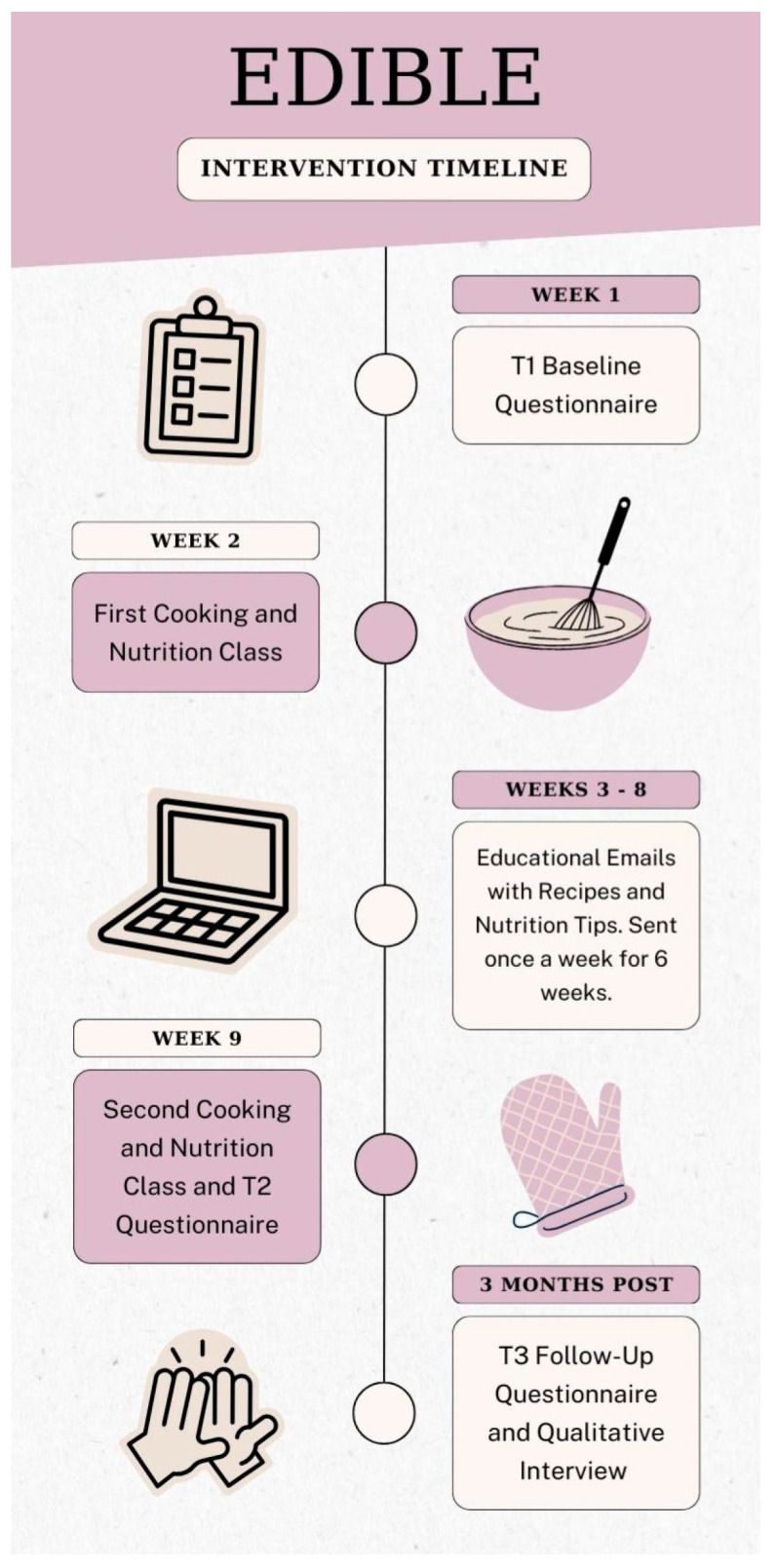
EDIBLE Intervention Timeline.

The EDIBLE intervention consisted of two 90 min interactive group workshops, which were held seven weeks apart. Each workshop was jointly led by a Wellness Chef and Registered Dietitian (RD), and had a maximum of 10 participants. Pairing an RD and a Chef in leadership roles is an innovative method for translating dietary guidelines into everyday practical skills [51]. Group education has also been shown to be an effective strategy for improving clinical outcomes in women with irritable bowel syndrome [52,53]. The initial class provided an introduction to GI side effects and their causes, as well as nutrition counseling specific to GI side effect management. Next, participants were led through a hands-on cooking session where they prepared three recipes specifically designed to address GI side effects. During the class, participants gained insights into the nutritional benefits and recommendations associated with each recipe, along with details about the ingredients. Additionally, participants received a comprehensive recipe package containing an ingredients list, step-by-step instructions, and nutrition tips to assist them during the session and reinforce their understanding afterward (see Supplemental File S1 data for an example recipe package). The participants were encouraged to ask questions, consider possible barriers to implementation, engage in discussions about potential solutions, and set personal goals. After the initial session, participants received emails weekly (for a total of six weeks). These emails reiterated essential information from the first class and provided a new recipe for participants to experiment with each week. The second class took place six weeks later. This class began with a recap of key learning points from the first class and weekly emails and fostered a group discussion about participants’ experiences trying the recipes. This was followed by an interactive cooking class featuring three additional recipes (see Figure 2 for the Intervention Timeline). All participants had the option to bring their spouse/partner/caregiver to observe the cooking demonstrations if they wished.

### 2.2. Participants and Procedures

Participants were recruited from the gynecology follow-up radiation clinics at the Princess Margaret Cancer Centre in Toronto, ON. Women aged 18 years or older with a diagnosis of non-metastatic cervical, endometrial, or vulvar cancer, who had completed pelvic RT ≥ 12 months prior, and were currently experiencing GI toxicity (identified by a score of <70 on the Short Inflammatory Bowel Disease Questionnaire (sIBDQ)) [54,55,56], were eligible to participate. Participants were also required to be proficient in English, have access to a kitchen, and express a willingness to cook. Women were excluded if they were currently receiving treatment; had inflammatory bowel disease, celiac disease, bowel obstructions or fissures; and/or had significant (i.e., anaphylaxis or multiple) food allergies.

Potential participants were identified from weekly generated clinic lists and chart reviews. Eligible participants were first approached by a member of their clinical circle of care during their follow-up visit and asked if they would be interested in learning more about the study. Those who expressed interest met with the Clinical Research Coordinator (CRC), who explained the study and asked them to complete the sIBDQ [54,55,56]. Women who reported a score of <70 on the sIBDQ were then invited to participate in the study and scheduled to attend the first class.

One week prior to the first class, participants were asked to sign the study consent form and complete the baseline questionnaires either online or on paper (T1). These measures were repeated immediately post-intervention (T2) and 3 months later (T3). Qualitative interviews were conducted in person or by telephone by a qualitative health researcher at T3 with a subsample of intervention participants to generate feedback on the program, including its format and structure, the delivery of information, the involvement of the Wellness Chef and RD, the value of the educational materials, the impact and impressions of the program, and suggestions for improvement. The interview guide was informed by the overarching aims of the study and designed to align with the administered questionnaires. All participants who completed at minimum T1 were invited to participate in a qualitative interview.

### 2.3. Study Outcomes

Demographic and Clinical Data: Basic demographic and clinical information were obtained through self-report and a chart review at baseline.

Primary Outcomes: The *feasibility* of the study intervention and study procedures was assessed by examining the following factors: (1) The accrual and retention rates, which were documented using a screening log with data for eligible and non-recruited patients, with the reasons for non-recruitment recorded when known. Reasons for attrition from the program were also documented. (2) The recorded outcomes, including the number of participants who completed the clinical outcome (primary and exploratory) assessments and documented rates of missing data. Feasibility was also informed by participants’ feedback regarding the adoption and implementation of the intervention during semi-structured qualitative interviews conducted at T3.

Qualitative interviews were also used to assess the *acceptability* of the intervention and inform future program refinement.

Secondary Outcomes (Exploratory Clinical Outcomes): Standardized questionnaires were administered in order to obtain a preliminary estimate of the impact of the EDIBLE intervention. Outcome measures for bowel and GI symptoms, knowledge, and self-efficacy were completed at baseline (T1), immediately post-intervention (T2), and at 3 months post intervention (T3).

(1) *Bowel and GI Symptoms*: Bowel symptoms were measured using the IBDQ [10,11,57,58]. The IBDQ is a disease-specific health-related QoL questionnaire which contains 32 questions pertaining to four dimensions (bowel, systemic, social, and emotional) [54] and has been shown to be valid, reliable, and responsive to change [54,55]. In addition, we administered the GI symptom subscale of the QLQ-EN24 [59].
(2) *Knowledge*: A 10-question multiple-choice nutrition and culinary knowledge questionnaire was developed and included items that were directly addressed during EDIBLE teaching sessions. Acquisition of knowledge is an important intermediate outcome in preparing survivors to self-manage [60].
(3) *Self-Efficacy:* A study-specific self-efficacy questionnaire was developed and included 5 items asking about participants’ confidence (scale 1–10) in their ability to manage bowel changes, to prepare food that will lessen bowel-related symptoms, and to eat a wide variety of foods, and their confidence in their knowledge about foods that improve or worsen bowel symptoms.

Qualitative interviews garnered further insight into the impact of the EDIBLE intervention on secondary outcomes.

### 2.4. Data Analysis

Patient characteristics were described with summary statistics and secondary outcomes were summarized at each time point. The capture rate for the secondary outcomes was calculated at each time point. The proportion of patients with complete capture (no missing data) was calculated at each time point as a proportion of the assessed participants. Mixed-effects models were fit to the outcomes of interest to examine changes from baseline to post-intervention and from post-intervention to follow-up, allowing for correlated measurements within participants using the nlme package in R [61,62]. Mixed-effects modeling of longitudinal data allows all available data to be used and does not require imputation [63]. To explore the extent to which the results may be biased due to losses to follow-up, baseline outcomes values were compared between those who did and did not complete all study assessments using the Wilcoxon rank sum test for continuous variables and chi-square tests of association for categorical variables. All statistical analyses were conducted using the R statistical programming language version 4.3.2 [62].

Qualitative interviews were audio recorded and transcribed verbatim. We employed a line-by-line coding approach to transcripts, creating initial codes and analytical concepts. This involved identifying unique terms used by participants themselves [64]. Initially, two researchers (C.P and M.R) independently coded the data and then held meetings to collaboratively develop, refine, and achieve consensus on key codes. Preliminary codes were those that directly aligned with project objectives, consistently appeared in individual interviews, and were discussed by multiple participants. Through this iterative process, a codebook was developed and refined. The final codebook was used to engage in a second round of coding in order to generate initial categories, progressing to second-level coding to construct preliminary themes, and supporting reflexive analytic memo writing [64]. Throughout this process, we utilized NVivo 12 software for data management, second-level coding, and generating thematic reports from interview quotations.

## 3. Results

**Figure 3:**
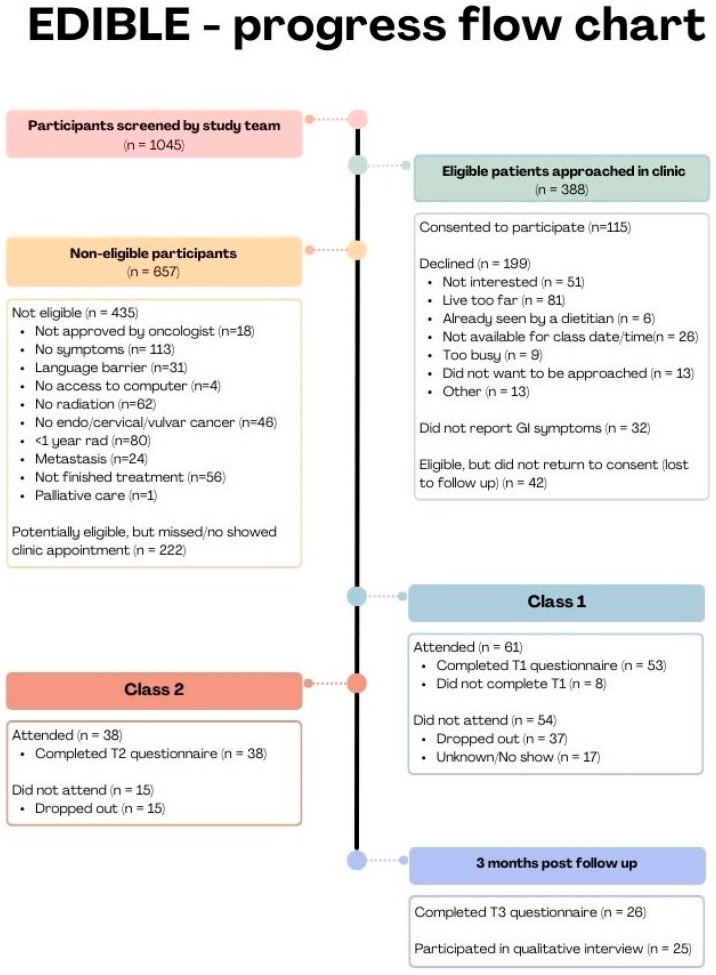
Flow Chart.

The study flow diagram is presented in Figure 3 and participants’ demographic and clinical characteristics are in presented in Table 1.

### 3.1. Feasibility

A total of *n* = 388 patients were eligible to participate and were approached during their clinic visit. Of these, 32 did not report GI symptoms and were deemed ineligible and *n* = 115 expressed interest and provided written consent (115/356, 32%), and *n* = 53 (46%) completed the baseline assessment (T1) and attended the first class (53/356, 15%). A total of 38/53 (72%) participants attended session two and completed the T2 assessment and 26/53 (49%) completed the three-month post-intervention follow-up assessment (T3). Of the 53 participants completing T1, 42 (79%) had complete data for all outcomes. The complete capture rate for the subsequent assessments was 32 (84%) at T2 and 24 (89%) at T3. Supplemental File S2, Table S1, provides capture rates for individual outcomes at each time point. These were generally very good (>90%).

There were four key themes developed from participants’ qualitative interviews pertaining to feasibility. These included *class length and program frequency; content clarity and style; ease of implementation, and program flexibility*. A summary of these themes is presented below, and a more fulsome analytic description of each of the themes, along with illustrative quotes, is summarized in Supplemental File S3, Table S6.

Most individuals believed that class duration and the frequency of in-class sessions were reasonable. Indeed, the class duration was described as “ideal”, allowing sufficient time for learning and allowing participants to personalize important nutrition information and culinary skills to meet their unique needs and challenges without being physically or mentally overwhelming. However, some suggested that having additional in-class sessions would have been beneficial for a more thorough immersion in nutrition and culinary practices. This was particularly relevant for those entering the program with limited nutrition knowledge and culinary skills. Both participants with and without prior nutrition and culinary expertise found the information and strategies acquired in the program easy to comprehend and apply at home. However, for some, limited access to technology imposed barriers to weekly email communications and program content. Implementing alternate methods for information delivery was critical to ensuring equitable access across participants.

### 3.2. Acceptability

Participants described different levels of side effect intensity; however, most participants—regardless of the intensity of their symptoms—explained that living with bowel-related issues (e.g., abdominal pain, constipation, fecal leakage, bloating, diarrhea) was “uncomfortable”, “painful”, and/or “embarrassing”. They further emphasized the ways in which these side effects imposed upon their day-to-day lives and activities, and negatively affected their overall QoL. All participants reinforced a strong belief in the link between food and health and felt that adjustments to their diet could help to reduce the severity and impact of their GI-related side effects. However, few knew how to adjust their diet, particularly since adherence to what is typically considered a “healthy” diet (e.g., a diet that is rich in whole grains, beans, vegetables, and fruit), could aggravate their GI symptoms. Participants hoped to receive guidance from “trusted experts” in the field, and personalized support with processes of dietary trial and error. While the exact motivations to join the EDIBLE program varied from participant to participant, they all rallied around an interest in regaining control over their bodies by addressing treatment-induced side effects and improving their general health. The extent to which the program was aligned with these motivations determined perceptions of acceptability.

Two overarching themes were developed from participants’ qualitative interviews pertaining to acceptability. These included satisfaction with the EDIBLE program and areas for program improvements. A summary of these themes is found below, and a more fulsome analytic description of each of their sub-themes, along with illustrative quotes, is summarized in Supplemental File S3, Table S7.

#### 3.2.1. Satisfaction with the EDIBLE Program

All participants expressed a high degree of satisfaction with the EDIBLE program. They particularly appreciated the small class sizes, which fostered a supportive and intimate environment conducive to personalized learning. Similarly, the program was praised for its person-centered approach, addressing individual dietary needs and GI side effects with tailored advice from trusted professionals. This individualized approach to program delivery helped reduce some of the uncertainty about dietary choices, creating a clearer path for behavior change. The in-person cooking demonstrations were especially appreciated, as they enhanced participants’ confidence and motivation to apply their newly acquired culinary skills at home. The group-based setting also provided valuable peer interaction, which further enriched the educational experience and helped normalize the challenges faced by cancer survivors living with GI-related side effects. Overall, participants found the program to be a significant source of support and empowerment in managing cancer-related GI side effects and dietary challenges.

#### 3.2.2. Areas for Program Improvement

Despite the high degree of satisfaction, feedback from participants also revealed several areas for improvement within the EDIBLE program, particularly with regard to enhancing its accessibility, tailoring its content, and expanding its focus. Participants emphasized several key recommendations for improving the EDIBLE program. They highlighted the importance of in-person sessions and expressed a desire for more, though they also acknowledged the need for flexible delivery options to accommodate those with accessibility challenges. To address varying levels of nutrition and culinary expertise among participants, a multi-tiered program structure was suggested, allowing individuals to enter the program and/or progress through different levels of the program based on their skills. Additionally, participants recommended systematically integrating personalized support into the program, such as incorporating more one-on-one consultations to ensure that all participants receive tailored advice, rather than just those who express a need for it. Finally, there was a call to expand the program’s focus to include other cancer-related side effects that often intersect with GI issues to enhance the program’s overall effectiveness. By addressing these areas, it was believed that the EDIBLE program would be better able to accommodate the diverse needs of its participants, enhance accessibility for some, and provide a more comprehensive approach to managing the wide-ranging and intersecting effects of cancer and its treatment.

### 3.3. Exploratory Clinical Outcomes

**Figure 4:**
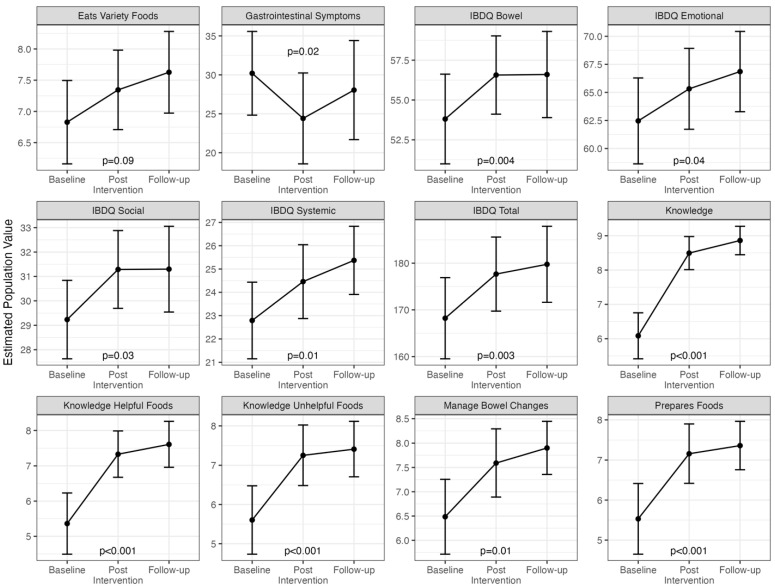
Estimated population averages across the three time points for each secondary outcome from mixed-effects models. The *p*-values are shown if the fixed-effect estimate of the change between baseline and post-intervention was significant at 0.05. No adjustments have been made for multiple comparisons.

Supplemental File S2 Table S2 describes the mean (sd) of each of the secondary outcomes across each assessment. Figure 4 illustrates the changes over time for each of the secondary variables. There were statistically significant improvements in bowel-related and GI symptoms, knowledge, and most of the self-efficacy outcomes. Full model estimates, including baseline values, changes from baseline to post-intervention and from post-intervention to follow-up and the random effects associated with participants are presented in Supplemental File S2, Table S3. We compared demographic characteristics and baseline outcome values for patients who did and did not complete all three assessments and did not find any statistically significant differences. These results are presented in Supplemental File S2, Tables S4 and S5.

Qualitative findings supported the quantitative results, reinforcing the positive impact of EDIBLE on participants’ knowledge, their capacity to self-manage (self-efficacy), and on reducing bowel-related and GI symptoms. Specifically, participants reported significant gains in their nutritional knowledge and culinary skills, which empowered them to manage GI symptoms with greater confidence and fostered post-program adherence to dietary changes. Many also described reduced fears around food, as the program clarified which dietary choices would alleviate symptoms, enabling eating habits that supported increased nutrient intake and avoidance of disruptive foods. Additionally, participants explained that they experienced alleviated bowel symptoms, improved energy levels, and a restored ability to engage in day-to-day activities like socializing and running errands without fear of GI issues. Even those who experienced minimal symptom relief valued the practical strategies and tools provided, noting enhanced cooking engagement and overall well-being. These findings underscore the broad utility of the EDIBLE program in addressing key challenges faced by gynecologic cancer survivors and highlight its capacity to improve both physical and psychosocial outcomes.

Detailed analytic descriptions and illustrative quotes for the three themes pertaining to perceptions of program utility and impact/clinical outcomes—knowledge and confidence, impact on diet and dietary habits, and impact to digestive irregularities, bowel symptoms and other side effects—are summarized in Supplemental File S3, Table S8.

## 4. Discussion

This pilot study was designed to determine the feasibility of a structured supported self-management dietary intervention (EDIBLE) for gynecologic cancer survivors experiencing RT-induced GI toxicity; to determine the acceptability and perceived utility of the intervention; and to obtain preliminary estimates on the effect of the intervention on bowel and GI symptoms, knowledge, and self-efficacy. Qualitative interviews supported strong perceptions of intervention feasibility; however, low accrual was attained, indicating that alternate formats for program delivery may be needed to make it more accessible. The acceptability of the EDIBLE intervention garnered especially high ratings on measures of satisfaction and utility, with program improvements largely rallying around a desire for increased in-class sessions and program expansion (i.e., creating tiered programs to permit additional opportunities for individual growth and development). While preliminary, favorable outcomes were observed at the three-month mark (T3); these included enhanced confidence in culinary practices, increased knowledge and skills for managing GI side effects, and improvements in bowel and GI symptoms. Overall, these positive results provide support for the acceptability and efficacy of the EDIBLE intervention, but also suggest that further refinement and evaluation of the EDIBLE intervention may be needed to promote improved recruitment and retention.

The recruitment rate of only 15% is similar to the recruitment rates for other dietary interventions recommended for cancer survivors [65,66,67]. The retention between class 1 and class 2 (72%) was moderate and is similar to that reported in a recent review of nutritional interventions in women with endometrial cancer [68]. The requirement to attend two in-person sessions may have affected participation and retention rates [69]. While our study reinforces the practical limitations of in-person attendance for some, the qualitative interviews also demonstrate an appreciation for in-person sessions, valued opportunities for connection and personalization through these sessions, and an expression of interest in more than two in-person classes. This underscores the importance of flexibility in program design and delivery, which will facilitate the optimization of accessibility across those with diverse needs and preferences. For instance, while alternate ways to deliver this intervention remotely/virtually are being explored in order to improve access for all, we also recognize the presence of a “digital divide” with regard to access and competency [70], as witnessed in the current study, and acknowledge that this needs to be considered in intervention programing to ensure equity of access for all who could benefit. Further, retention methods such as compensation to cover costs related to attendance, and tailoring of materials to meet individual needs, have been shown to boost retention in dietary trials [69] and should be considered for future research. Notwithstanding low recruitment and moderate retention, qualitative feedback pertaining to class length and program frequency, content clarity and style, ease of implementation, and program flexibility supported strong perceptions of intervention feasibility from participants’ perspectives.

The EDIBLE intervention achieved a high degree of acceptability among qualitative interview participants, emphasizing their favorable response and readiness to participate in a dietary intervention aimed at enhancing side effect management. Current literature indicates that modifying dietary practices is among the most consistently reported self-management strategy employed by cancer survivors to address bowel-related and GI symptoms [71,72]. This is consistent with the experiences of EDIBLE participants who emphasized efforts to manage their symptoms with food, and perceived this to be a desirable and feasible strategy for symptom management. Qualitative findings from our study also confirmed that general nutrition advice and diet modification guidance provided for cancer survivors often falls short in addressing the specific dietary challenges and needs of individuals coping with GI side effects [71,72]. For some of the participants, this lack of guidance caused them to fear food or even avoid it altogether, which could lead to a risk of malnutrition in a population that is already predisposed to poor nutrient uptake, which can worsen prognosis, overall health, and QoL [73]. This reinforces the need for interventions aimed at assisting survivors in making suitable dietary modifications for the management of bowel and GI symptoms. EDIBLE was perceived as especially valuable because it provided a more tailored approach that considered the general sensitivities and tolerances of cancer survivors who have received pelvic RT, while also acknowledging the need for person-centered approaches that consider the diverse nature of individuals’ post-treatment experiences, GI side effects, and dietary preferences. Focusing on the taste, individual preferences, and therapeutic benefits of certain foods, the EDIBLE intervention supported not only the physiological needs of survivors but also the social, emotional, and cultural relationships that people have with food, filling an important gap in supportive care [74]. Opportunities to learn and implement new culinary strategies in the execution of nutrition information were emphasized as being foundational to many participants’ retention of nutrition information and feasibility of implementation. This underscores the value of adding an interactive cooking component to nutrition education for cancer survivors, and ultimately reinforces the advantages of utilizing cooking classes as a platform for implementing dietary interventions [75,76,77,78]. It also illuminates the instrumental role of instructors in building motivation, confidence, and competence in culinary practices, ultimately facilitating greater participation in meal preparation and enhancing consumption of foods that meet the nutritional needs of cancer survivors with GI side effects.

Evidence of outcome improvements associated with the EDIBLE intervention at three months, including enhanced confidence in culinary practices, increased knowledge and skills with regard to the management of GI side effects (self-efficacy), and improvements in bowel and GI symptoms, are encouraging indicators of the impact of the EDIBLE intervention. Qualitative findings supported these results and revealed that newfound dietary and culinary knowledge and confidence improved motivation for long-term adherence and, for some, provided an improved sense of empowerment and self-efficacy. Development of self-efficacy at a time when patients are experiencing a loss of bodily autonomy is particularly important for rebuilding their sense of control and well-being [79]. Indeed, while some qualitative participants reported only marginal or no improvements to their GI symptoms, improved dietary knowledge and culinary skills facilitated improved nutrition intake without further aggravating GI symptoms, which participants acknowledged as instrumental in improving their overall sense of health, well-being, and QoL.

### Strengths and Limitations

As the first study to develop and systematically test a dietary self-management support program addressing radiation treatment-induced GI toxicity, our project may help to fill a significant gap in survivorship care. By addressing a previously unmet need in this population, this pilot study lays the foundation for further refinement and larger-scale testing of dietary self-management interventions in oncology care and for other GI diseases. This study provides useful information on the feasibility, acceptability, and impact of a culinary nutrition intervention for the management of GI toxicity post pelvic RT. The use of qualitative methods offered a depth of understanding and nuance in discussions about interventions’ feasibility, acceptability, and impact that will be critical for refining the intervention and improving overall implementation. In addition to interviewing those who completed the EDIBLE intervention, we also welcomed participation from those who did not complete the intervention (e.g., attended class 1 but not class 2). These interviews helped to provide clarity of understanding pertaining to program barriers underpinning intervention attrition. While the results of this intervention are promising, there are several limitations to consider. Although the sample size was sufficient for a pilot study focused on acceptability and feasibility, the observed impact should be viewed as preliminary and interpreted with caution. Additionally, the absence of a control group leaves room for other potential explanations for the improvements in GI symptoms, including the passage of time and/or participation in other rehabilitative programs, including exercise programs. A randomized controlled trial would offer more definitive insights into the program’s direct effects. Lastly, while group-based sessions enhanced the overall acceptability of the intervention and are likely to reduce cost [76,80], a cost-effectiveness analysis was not performed and may be needed to further measure program feasibility and scalability.

## 5. Conclusions

GI toxicity can lead to malnutrition as well as diminished well-being and overall quality of life among gynecologic cancer survivors. While preliminary, the results demonstrate that the EDIBLE intervention is an acceptable approach for addressing GI toxicity that resulted in statistically and clinically significant improvements in GI symptom management for gynecologic cancer survivors post pelvic RT. However, moderate recruitment and retention rates highlight the need for program refinement, such as alternative delivery methods to improve accessibility. Additionally, while qualitative data provided valuable insights into participant experiences, future research, including a randomized controlled trial and cost-effectiveness analyses, is required to confirm the intervention’s effectiveness and scalability.

## Figures and Tables

**Table 1:** Participant characteristics.

Participants	*n* = 53
**Age**	
Mean (sd)	63.1 (10.5)
Median (Min, Max)	64 (38, 91)
**Marital Status**	
Married/Common law	17 (32%)
Divorced/Separated	12 (23%)
Widowed	9 (17%)
Single, never married	15 (28%)
**Race and Ethnicity**	
Black	2 (4%)
East Asian	6 (11%)
Indigenous	1 (2%)
Latino	2 (4%)
South Asian	1 (2%)
Southeast Asian	1 (2%)
West Asian	1 (2%)
White	36 (68%)
Other	1 (2%)
Missing	2 (4%)
**Cancer Type**	
Cervical	9 (17%)
Endometrial	40 (75%)
Ovarian	1 (2%)
Undisclosed	3 (6%)
**Treatment Type**	
Radiation	53 (100%)
Surgery	41 (77%)
Chemotherapy	29 (55%)

## Data Availability

The datasets presented in this article are not readily available because REB requirements. Requests to access the datasets should be directed to the corresponding author (J.M.J.).

## Supplementary Materials

The following supporting information can be downloaded at https://www.mdpi.com/article/10.3390/nu16234227/s1, File S1: Example Recipe Package; File S2: Quantitative Data Tables, Table S1. Data capture rates for secondary outcomes as a proportion of assessments completed for each outcome; Table S2. Mean (sd) of secondary outcomes at each time point; Table S3. Results of mixed effects models estimating change across assessment periods; Table S4. Demographic comparisons of participants who did and did not complete all three study assessments; Table S5. Comparisons of baseline outcome values between patients who did and did not complete all study assessments; File S3: Qualitative Data Tables, Table S6. Qualitative Table for Feasibility; Table S7. Qualitative Table for Acceptability; Table S8. Qualitative Table for Clinical Outcomes.
