# Supplementary material for: Eliminating Digestive Irregularities Caused by Late Effects: A Pilot Study of an Innovative Culinary Nutrition Intervention for Reducing Gastrointestinal Toxicity in Gynecologic Cancer Patients Who Have Undergone Pelvic Radiotherapy
Source: Nutrients. 2024 Dec 6;16(23):4227. doi: 10.3390/nu16234227 (PMC11644450; doi:10.3390/nu16234227)
Supplement: Supplementary file 1 [file nutrients-16-04227-s001.zip › S1 EDB Class 1 Recipes (1).pdf]

EDIBLE | Eliminating Digestive Irregularities caused By Late Effects of abdominopelvic radiation

Supplemental File 1 (S1): Example Recipe Package

Welcome to the EDIBLE program!

This program is designed to pair nutrition education with culinary instruction to support people affected by cervical or endometrial cancer cope with their digestive side effects.

Classes feature:

- Hands-on cooking alongside Wellness Chef Geremy Capone and Registered Dietitian Stephanie Gladman
- Learning in a supportive group setting about nutritional benefits of the foods you prepare and how they can help your symptoms
- Nutritional and tasty food samples for you to try

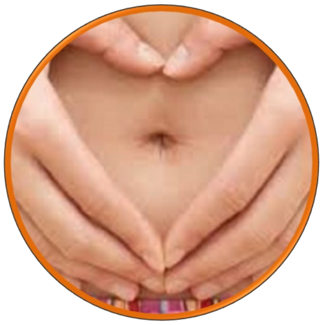

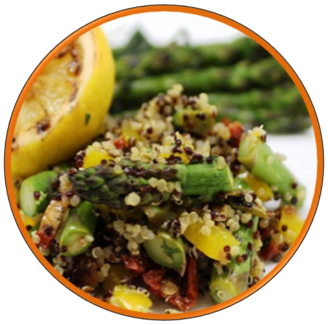

Location: ELLICSR: Health, Wellness & Cancer Survivorship Centre
Toronto General Hospital, Basement level, BCS-021

Contact: Email edible@uhnresearch.ca for more information

We hope you will enjoy the program and be sure to look out for our weekly emails with more cooking tips and recipes!

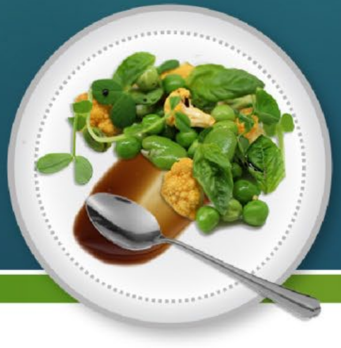

Tomato Basil Soup

Skill Level: *
Preparation Time: 25 minutes
Total Time: 60 minutes
Servings: 4
Cost Per Serving: $2.38

Ingredients

12	Fresh Plum Tomatoes (or 2 large cans of whole peeled tomatoes)
1 cup	Fresh Basil
1 cup	Carrot, grated
2 cloves	Garlic (optional)
2 cups	Vegetable or Chicken Stock
2 tbsp	Olive Oil
½ tsp	Sea Salt and Freshly Ground Black Pepper

Nutrition Notes

- Tomatoes are rich in beta carotene, an antioxidant that is converted into vitamin A in the body. Vitamin A helps protect the eyes from free radicals, compounds that can damage cells, and is needed for a healthy immune system.

- Tomatoes also contain a powerful antioxidant called lycopene. Lycopene helps prevent free radical damage and may help fight cancer. Lycopene may also help lower the risk of heart disease. Cooking tomatoes more than doubles the amount of lycopene in them.

- This soup freezes well, so it can be made ahead in large batches and frozen in individual containers. Cook once and eat several meals to save energy and time!

Directions

1. If using fresh plum tomatoes, bring a large pot of water to a boil. Score the bottom of each tomato with a small cross.
2. Put a bowl of ice water to the side.
3. Add the tomatoes to the boiling water. Cook for about 60 seconds and then remove and place directly into the ice water. This will allow you the remove the skins easily. Try and squeeze some of the seeds out too.
4. Add all the tomatoes, basil, carrot, garlic, stock, olive oil, salt and pepper into a large sauce pot over medium high heat.
5. Stir gently and simmer for 30 minutes.
6. Either blend until pureed or pass through a food mill to remove even more of the seeds.
7. Serve with some fresh basil.

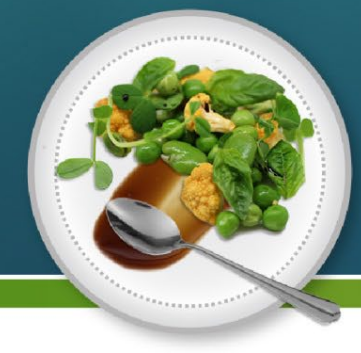

Fish Cooked in Parchment with Pineapple Couscous

Skill Level: *

Preparation Time: 20 minutes

Total Time: 40 minutes

Servings: 4

Cost Per Serving: $5.43

Ingredients

4 – 4oz	Fish Filets (Steelhead Trout, Pacific Salmon or Arctic Char)
1 cup	Couscous
1 cup	Warm Water or Stock
1 cup	Pineapple, small dice
1 cup	Red Pepper, small dice
½ tbsp	Extra Virgin Olive Oil
½ tsp	Sea Salt and Freshly Ground Black Pepper

Marinade Ingredients

1	Lime, juice and zest
¼ cup	Cilantro or any herb
1 tbsp	Ginger, grated
½ tbsp	Extra Virgin Olive Oil or Sesame Oil

Nutrition Notes

- Oily fish such as trout and salmon are excellent sources of brain-boosting omega-3 fatty acids. Diets rich in omega-3 fatty acids may play a role in preventing depression and help keep your memory sharp as you get older. Omega-3s have also been shown to improve brain function in older adults.

- Omega-3s lower inflammation, a risk factor for cancer.

- Choose at least 2 servings of oily fish each week to get the benefits from omega-3s. One serving of fish is 3 ounces or the size of a deck of cards.

Directions

1. Preheat the oven to 375 degrees F.
2. Place your couscous, pineapple, and red peppers, in a bowl, add your hot water, cover with plastic wrap and let sit for 5 minutes until liquid has absorbed. Once done, fluff with a fork.
3. Tear off a large piece of parchment paper for each piece of fish. Fold in half. Add some of your couscous in the middle of one half of the paper. Add a piece of fish on top.
4. Mix all the marinade ingredients together in a bowl. Pour over the fish filets.
5. Add any extra vegetables or seasonings that you enjoy on top.
6. Seal the parchment pouches by folding over the edges to create a tight seal.
7. Place on a baking pan, and cook for about 12 - 15 minutes.
8. Carefully open the parchment to release the steam, make sure the fish is cooked through and flakes easily.

Coconut, Banana & Cherry Ice Cream

Skill Level: *
Preparation Time: 15 minutes
Total Time: 45 minutes
Servings: 4
Cost Per Serving: $0.88

Ingredients

2	Ripe Bananas (peel and freeze)
2 tbsp	Maple Syrup or Honey
½ tsp	Ground Cinnamon
½ cup	Coconut Milk (light or from scratch)
½ cup	Fresh and Frozen Cherries

Coconut Milk Ingredients

1 cup	Grated Fresh Coconut or Unsweetened Shredded Coconut
2 cups	Hot Water

Nutrition Notes

- Shredded coconut is high in fibre, but coconut milk has the fibre removed from it. Coconut milk is high in fat, so look for a lighter version. If you are making your own coconut milk, make sure you remove any fat that separates (it will be on the top of your coconut milk).

- Bananas are easy to digest and are one of the few fruits recommended to try if you have diarrhea. Although bananas are a good source of fibre, this fibre is in the form of pectin, a soluble fibre that helps slow down the movement of food through your digestive system.

- Cherries are rich in antioxidants called anthocyanins that may help reduce inflammation in the joints. They also contain quercetin, an antioxidant that may lower blood pressure by helping to keep blood vessels relaxed and flexible.

Directions

1. Thaw out the frozen bananas at room temperature for about 5 minutes. Add bananas to a food processor with maple syrup, cherries, and coconut milk and blend until smooth. Serve immediately or freeze until ready to use.
2. Enjoy!

3. * To make your own coconut milk, combine 1 cup of grated coconut with 2 cups of hot water in a blender. Let it sit for two minutes. Blend on high for about 2 minutes. Strain through a cheese cloth or a clean dish towel and squeeze all the pulp to ring out any last bit of water. Transfer the coconut milk into a tight fitted jar and store in the fridge. Fat will separate out of the coconut milk and form a layer on top. Remove this and store in an airtight container. You can use the coconut oil in recipes in small amounts.
