# Supplementary material for: Eliminating Digestive Irregularities Caused by Late Effects: A Pilot Study of an Innovative Culinary Nutrition Intervention for Reducing Gastrointestinal Toxicity in Gynecologic Cancer Patients Who Have Undergone Pelvic Radiotherapy
Source: Nutrients. 2024 Dec 6;16(23):4227. doi: 10.3390/nu16234227 (PMC11644450; doi:10.3390/nu16234227)
Supplement: Supplementary file 1 [file nutrients-16-04227-s001.zip › S2 Quantitative Data Tables.pdf]

Supplemental File 2 (S2): Quantitative Data Tables

S2. Table A. Data capture rates for secondary outcomes as a proportion of assessments completed for each outcome.

| **Outcome** | **Baseline T1 (n=53)** | **Post-Intervention T2 (n=38)** | **Follow-Up T3 (n=26)** |
|---|---|---|---|
| IBDQ Total Score | 53 (100) | 38 (100) | 26 (100) |
| IBDQ Bowel Score | 53 (100) | 38 (100) | 26 (100) |
| IBDQ Systemic Score | 53 (100) | 38 (100) | 26 (100) |
| IBDQ Emotional Score | 53 (100) | 38 (100) | 26 (100) |
| IBDQ Social Score | 52 (98) | 38 (100) | 25 (96) |
| Self Efficacy manage bowel changes | 52 (98) | 38 (100) | 26 (100) |
| Self Efficacy know foods improves bowel | 48 (91) | 38 (100) | 26 (100) |
| Self Efficacy know foods worsen bowel | 49 (92) | 33 (87) | 25 (96) |
| Self Efficacy eat wide variety foods | 52 (98) | 38 (100) | 26 (100) |
| Self Efficacy prepare foods lessen bowel | 51 (96) | 38 (100) | 26 (100) |
| QLQ-EN24 Gastrointestinal Symptoms | 51 (96) | 38 (100) | 25 (96) |
| Knowledge Score | 48 (91) | 37 (97) | 26 (100) |

S2. Table B. Mean (sd) of secondary outcomes at each time point.

| Outcome | Baseline T1 (n=53) | Post-Intervention T2 (n=38) | Follow-Up T3 (n=26) |
|---|---|---|---|
| IBDQ Total Score | 168.2 (32.3) | 177.2 (27.1) | 179.9 (24.0) |
| IBDQ Bowel Score | 53.8 (10.5) | 56.4 (8.7) | 56.6 (7.4) |
| IBDQ Systemic Score | 22.8 (6.1) | 24.2 (5.4) | 25.5 (4.8) |
| IBDQ Emotional Score | 62.5 (14.2) | 65.4 (12.1) | 67.1 (10.9) |
| IBDQ Social Score | 29.3 (5.9) | 31.2 (5.2) | 31.1 (5.5) |
| Self Efficacy manage bowel changes | 6.5 (2.8) | 7.5 (2.3) | 7.9 (1.5) |
| Self Efficacy know foods improves bowel | 5.3 (3.1) | 7.3 (2.1) | 7.6 (1.7) |
| Self Efficacy know foods worsen bowel | 5.6 (3.2) | 7.3 (2.3) | 7.6 (1.8) |
| Self Efficacy eat wide variety foods | 6.8 (2.5) | 7.2 (2.2) | 7.4 (2.0) |
| Self Efficacy prepare foods lessen bowel | 5.6 (3.2) | 7.1 (2.4) | 7.3 (1.7) |
| Knowledge Score | 6.1 (2.4) | 8.5 (1.6) | 9.0 (1.2) |
| QLQ-EN24 Gastrointestinal Symptoms | 29.7 (19.0) | 24.6 (19.9) | 29.1 (18.8) |

S2. Table C. Results of mixed effects models estimating change across assessment periods. Baseline refers to the average value prior to the intervention. Intervention Change refers to the estimated change from baseline to post-intervention (T1 to T2). Follow-up Change refers to the estimated change from post-intervention to the follow-up assessment (T2 to T3). Random effects are estimates of the variability within the population, expressed in standard deviations.

| | Estimate | 95% CI | p-value | Random Effect (SD) |
|---|---|---|---|---|
| **IBDQ Total Score** | | | | |
| Baseline | 168.21 | (159.36, 177.07) | | 31.56 |
| Intervention Change | 9.45 | (3.39, 15.51) | 0.003 | 17.04 |
| Follow-up Change | 2.09 | (-3.27, 7.45) | 0.44 | 10.84 |
| Residual | | | | 6.64 |
| **IBDQ Bowel Score** | | | | |
| Baseline | 53.81 | (50.94, 56.68) | | 10.15 |
| Intervention Change | 2.76 | (0.93, 4.59) | 0.004 | 4.76 |
| Follow-up Change | 0.03 | (-2.57, 2.63) | 0.98 | 6.25 |
| Residual | | | | 2.50 |
| **IBDQ Systemic Score** | | | | |
| Baseline | 22.79 | (21.12, 24.47) | | 5.92 |
| Intervention Change | 1.67 | (0.38, 2.95) | 0.01 | 3.54 |
| Follow-up Change | 0.91 | (-0.38, 2.20) | 0.16 | 2.92 |
| Residual | | | | 1.46 |
| **IBDQ Emotional Score** | | | | |
| Baseline | 62.46 | (58.56, 66.36) | | 13.92 |
| Intervention Change | 2.86 | (0.11, 5.61) | 0.04 | 7.79 |
| Follow-up Change | 1.54 | (-0.55, 3.63) | 0.15 | 3.65 |
| Residual | | | | 2.89 |
| **IBDQ Social Score** | | | | |
| Baseline | 29.23 | (27.60, 30.87) | | 5.68 |
| Intervention Change | 2.05 | (0.20, 3.90) | 0.03 | 5.54 |
| Follow-up Change | 0.01 | (-1.33, 1.35) | 0.98 | 2.52 |
| Residual | | | | 1.64 |
| **Self Efficacy manage bowel changes** | | | | |
| Baseline | 6.48 | (5.70, 7.27) | | 2.74 |
| Intervention Change | 1.11 | (0.24, 1.97) | 0.01 | 2.63 |
| Follow-up Change | 0.31 | (-0.36, 0.98) | 0.36 | 1.57 |
| Residual | | | | 0.76 |
| **Self Efficacy know foods improves bowel** | | | | |
| Baseline | 5.36 | (4.47, 6.25) | | 3.01 |
| Intervention Change | 1.97 | (1.14, 2.80) | <0.001 | 2.41 |
| Follow-up Change | 0.28 | (-0.37, 0.93) | 0.40 | 1.36 |
| Residual | | | | 0.79 |
| **Self Efficacy know foods worsen bowel** | | | | |
| Baseline | 5.60 | (4.71, 6.50) | | 3.04 |
| Intervention Change | 1.65 | (0.85, 2.44) | <0.001 | 2.09 |
| Follow-up Change | 0.16 | (-0.81, 1.13) | 0.74 | 2.34 |

| | Estimate | 95% CI | p-value | Random Effect (SD) |
|---|---|---|---|---|
| Residual | | | | 0.83 |
| **Self Efficacy eat wide variety foods** | | | | |
| Baseline | 6.83 | (6.15, 7.51) | | 2.37 |
| Intervention Change | 0.52 | (-0.08, 1.12) | 0.09 | 1.64 |
| Follow-up Change | 0.28 | (-0.34, 0.91) | 0.37 | 1.38 |
| Residual | | | | 0.69 |
| **Self Efficacy prepare foods lessen bowel** | | | | |
| Baseline | 5.53 | (4.63, 6.43) | | 3.15 |
| Intervention Change | 1.63 | (0.76, 2.50) | <0.001 | 2.64 |
| Follow-up Change | 0.20 | (-0.36, 0.77) | 0.48 | 1.19 |
| Residual | | | | 0.73 |
| **QLQ-EN24 Gastrointestinal Symptoms** | | | | |
| Baseline | 30.20 | (24.71, 35.69) | | 19.03 |
| Intervention Change | -5.79 | (-10.72, -0.86) | 0.02 | 13.15 |
| Follow-up Change | 3.64 | (-1.55, 8.82) | 0.17 | 11.33 |
| Residual | | | | 5.46 |
| **Knowledge Score** | | | | |
| Baseline | 6.08 | (5.40, 6.77) | | 2.31 |
| Intervention Change | 2.41 | (1.72, 3.10) | <0.001 | 2.06 |
| Follow-up Change | 0.37 | (-0.15, 0.89) | 0.16 | 1.17 |
| Residual | | | | 0.60 |

S2. Table D. Demographic comparisons of participants who did and did not complete all three study assessments. Statistical tests run were the Wilcoxon Rank Sum (WRS), Fisher's Exact test (FE) or the Chi-square test (CS)

| | Completed T3 (n=26) | Lost to Follow-up (n=27) | p-value | Statistical Test |
|---|---|---|---|---|
| **Age** | | | 0.30 | WRS |
| Mean (sd) | 62.0 (10.4) | 64.0 (10.8) | | |
| Median (Min,Max) | 62 (44, 87) | 66.5 (38.0, 91.0) | | |
| Missing | 0 | 1 | | |
| **Marital_Status** | | | 0.81 | FE |
| Married/Common-law | 8 (31) | 9 (33) | | |
| Divorced/Separated | 5 (19) | 7 (26) | | |
| Widowed | 4 (15) | 5 (19) | | |
| Single, never married | 9 (35) | 6 (22) | | |
| **Ethnicity** | | | 0.67 | FE |
| Black | 0 (0) | 2 (8) | | |
| East Asian | 3 (12) | 3 (12) | | |
| Indigenous | 0(0) | 1(2) | | |
| Latino | 1 (4) | 1 (4) | | |
| South Asian | 0 (0) | 1 (4) | | |
| Southeast Asian | 1 (4) | 0 (0) | | |
| West Asian | 0 (0) | 1 (4) | | |
| White | 20 (80) | 16 (64) | | |
| Other | 0 (0) | 1 (4) | | |
| Missing | 1 | 2 | | |
| **Ethnicity** | | | 0.67 | FE |
| White | 20 (80) | 16 (64) | | |
| Asian | 3 (12) | 3 (12) | | |
| South Asian | 0 (0) | 1 (4) | | |
| Southeast Asian | 1 (4) | 0 (0) | | |
| West Asian | 0 (0) | 1 (4) | | |
| Black | 0 (0) | 2 (8) | | |
| Latino | 1 (4) | 1 (4) | | |
| Other | 0 (0) | 1 (4) | | |
| Missing | 1 | 2 | | |
| **Cancer Type** | | | 0.52 | FE |
| Cervical | 6 (23) | 3 (11) | | |
| Endometrial | 18 (69) | 22 (81) | | |
| Other | 2 (8) | 2 (7) | | |
| **Treatment: Radiation** | | | | No test |
| Radiation | 26 (100) | 27 (100) | | |
| **Treatment: Surgery** | | | 0.80 | CS |
| No | 5 (19) | 7 (26) | | |
| Yes | 21 (81) | 20 (74) | | |
| **Treatment: Chemotherapy** | | | 0.15 | CS |
| No | 8 (32) | 15 (56) | | |
| Yes | 17 (68) | 12 (44) | | |

| | Completed T3 (n=26) | Lost to Follow-up (n=27) | p-value | Statistical Test |
|---|---|---|---|---|
| Missing | 1 | 0 | | |

S2. Table E. Comparisons of baseline outcome values between patients who did and did not complete all study assessments. Tests of differences in median value were conducted using the Wilcoxon Rank Sum test and the corresponding p-values are reported.

| **Outcome** | **Completed T3 (n=26)** | **Lost to Follow-up (n=27)** | **p-value** |
|---|---|---|---|
| IBDQ Total Score | | | 0.96 |
| mean (sd) | 167.7 (31.7) | 168.7 (33.4) | |
| median (min, max) | 174.9 (83.0, 207.0) | 173.4 (103.2, 222.0) | |
| IBDQ Bowel Score | | | 0.49 |
| mean (sd) | 53.2 (9.4) | 54.4 (11.5) | |
| median (min, max) | 54.8 (36.0, 67.0) | 56 (30, 70) | |
| IBDQ Systemic Score | | | 0.75 |
| mean (sd) | 23.0 (6.4) | 22.6 (5.9) | |
| median (min, max) | 23 (11, 34) | 23 (13, 34) | |
| IBDQ Emotiol Score | | | 0.86 |
| mean (sd) | 62.9 (13.7) | 62.1 (14.9) | |
| median (min, max) | 67 (26, 78) | 64 (31, 84) | |
| IBDQ Social Score | | | 0.56 |
| mean (sd) | 28.6 (6.8) | 29.9 (5.0) | |
| median (min, max) | 30.5 (7.0, 35.0) | 31 (17, 35) | |
| missing | 0 | 1 | |
| Self Efficacy mage bowel changes | | | 0.4 |
| mean (sd) | 6.0 (3.1) | 6.9 (2.5) | |
| median (min, max) | 7 (0, 10) | 7 (2, 10) | |
| missing | 0 | 1 | |
| Self Efficacy know foods improves bowel | | | 0.75 |
| mean (sd) | 5.2 (2.9) | 5.4 (3.4) | |
| median (min, max) | 5 (0, 10) | 6 (0, 10) | |
| missing | 3 | 2 | |
| Self Efficacy know foods worsen bowel | | | 0.28 |
| mean (sd) | 6.2 (2.5) | 5.0 (3.6) | |
| median (min, max) | 6 (1, 10) | 5 (0, 10) | |
| missing | 2 | 2 | |
| Self Efficacy eat wide variety foods | | | 0.36 |
| mean (sd) | 6.4 (2.7) | 7.2 (2.2) | |
| median (min, max) | 6.5 (0.0, 10.0) | 7 (3, 10) | |
| missing | 0 | 1 | |
| Self Efficacy prepare foods lessen bowel | | | 0.36 |
| mean (sd) | 5.2 (2.9) | 5.9 (3.4) | |
| median (min, max) | 5 (1, 10) | 7 (0, 10) | |
| missing | 1 | 1 | |

| | | | |
|---|---|---|---|
| QLQ-EN24 Gastrointestil Symptoms | | | 0.28 |
| mean (sd) | 32.5 (17.7) | 26.9 (20.2) | |
| median (min, max) | 33.3 (0.0, 60.0) | 26.7 (0.0, 66.7) | |
| missing | 1 | 1 | |
| Knowledge Score | | | 0.094 |
| mean (sd) | 6.5 (2.8) | 5.7 (1.9) | |
| median (min, max) | 7 (0, 10) | 6 (1, 9) | |
| missing | 2 | 3 | |
