# Supplementary material for: Eliminating Digestive Irregularities Caused by Late Effects: A Pilot Study of an Innovative Culinary Nutrition Intervention for Reducing Gastrointestinal Toxicity in Gynecologic Cancer Patients Who Have Undergone Pelvic Radiotherapy
Source: Nutrients. 2024 Dec 6;16(23):4227. doi: 10.3390/nu16234227 (PMC11644450; doi:10.3390/nu16234227)
Supplement: Supplementary file 1 [file nutrients-16-04227-s001.zip › S3 Qualitative Data Tables.pdf]

Supplemental File 3 (S3): Qualitative Data Tables

S3. Table F. Qualitative Table for Feasibility

Theme	Analytic note	Illustrative quotes
Class length and program frequency	All participants felt that class length (90-minute in-person class) was ideal. Many stated that if classes were shorter in duration, there would be insufficient time for experiential learning and personalization of information through question and answer sessions with the program instructors. Many explained that if classes were any longer than 90 minutes, there would be a risk of information overload, difficulty retaining information, and/or that sessions may become too physically strenuous/challenging, particularly in light of their treatment side effects (including bowel issues, as well as pain and fatigue).<br><br>Most explained that two in-class sessions, paired with weekly videos and written email communications was appropriate in terms of frequency of outreach, and volume of information. Although, several participants also expressed an interest in attending additional in-person classes, if appropriately spaced out. The delivery of content was described by most as "abundant" and "informative", but without feeling overwhelming.	"[Timing of the in-class sessions] was good for me. Like, it seems enough time [to] give me information, do some cooking, answer questions. I think shorter, it would be too rushed."<br><br>"I thought the length was good because if it was too long then I probably wouldn't retain the information."<br><br>"I think [the duration of the class] is just about right. I don't think a lot of people could handle too much more. I think it would be nice, but I don't think we physically could [do it]."<br><br>"I think if they had another class later on in the year, I think everyone would attend."
Content clarity and style	Nearly all participants felt that the program content was clear, jargon-free, easy to follow, and well suited to meet their unique informational needs.<br><br>Participants also appreciated having access to different mediums of information (e.g., in-person and interactive sessions, written information, and videos), accessible at different time points, and from different locations. Some even expressed that diversity in content delivery was helpful in reaching different types of learners (e.g., visual learners), and that this supported an	"So [the information] was presented in a way that was easy to understand and apply. So, it wasn't too complicated […] it was all very useful information, you could tell it was very specific [to our needs]"<br><br>"I am a visual learner, so I can read something and I can do it, but if I get the chance to see it, I can do it much easier…so, I really found that helpful"

	element of inclusivity and personalization to the course.	
Ease of implementation	Participants across different culinary and nutrition skill levels found that recipes and culinary techniques taught in the EDIBLE program were easily implemented into their everyday practice, and that delivery of follow-up content (emails and videos) helped to keep participants engaged between in-person sessions. Ease of implementation within the home was further supported by the provision of recipes that were not overly complicated or complex, were cost-considerate, and contained ingredients that participants perceived as being accessible.<br><br>In-person sessions seemed to be particularly helpful in supporting those with less pre-intervention cooking experience and nutritional knowledge. For these individuals, watching and making recipes in a group setting made cooking more approachable.	"I like that the recipes are not too many ingredients and not too complicated and its stuff that I would actually do which is the main thing because I have so many cookbooks that I buy and I just look at them and I don't do anything with them."<br><br>"The good thing about the weekly emails is that keeps you engaged in the process. And I think that is important."<br><br>"[I learned] that it does not have to be expensive to eat healthy, and well, and you don't have to use some special ingredients. What they used were some everyday things that are easy to find at most grocery stores and markets."<br><br>"Yes, the demonstrations were perfect. For people with less cooking experience or who find cooking intimidating, it shows you what you are doing and you taste after. It shows you that it's simple and you don't have to make a big deal out of it, like I do."
Program flexibility	The design of the EDIBLE program is such that other than the two in-person sessions, the program is largely virtual, requiring email and computer/smartphone access as well as access to the internet. A few participants mentioned that a lack of access to technology affected their ability to look at content provided through email. For those who expressed access issues to the program instructors, accommodations were made where possible, such as printing informational content to mail to the participant's home. Other participants explained that virtual content was printed and provided during in-person classes, and/or time was arranged to pick up printed materials during participants' medical appointments.	"Well, I don't have a computer at the moment, so I got all the material printed out to take home with me, since I didn't have access to an email."

S3. Table G. Qualitative Table for Acceptability

Theme	Analytic note	Illustrative quotes
**Satisfaction with the EDIBLE Program**	See sub-themes below	See sub-themes below
Sub-theme: Class size	All participants appreciated small class sizes (< n=10) as it fostered a more intimate setting for connection with and support from other cancer survivors dealing with similar side effects. Small class sizes also afforded the appropriate space and time to maximize experiential learning and personalize (through discussions with the program instructors) the guidance, nutrition information, and cooking techniques to meet individual program participant needs.<br><br>Quality of life and emotional well-being were also enhanced by the interactive nature of more intimate class sizes, which fostered a sense of community and shared experience. For some, participating in the cooking demonstrations became more than a practical knowledge and skill-building exercise; it provided a supportive and uplifting environment where participants could share their challenges and successes, contributing to a sense of normalcy, community, and joy.	"I liked that it was a small amount of people. The intimacy of it was excellent, and there was lots of room to ask questions and there was room for other people to comment and to learn from each other's experiences."<br><br>"You just feel that you've been so exposed with everything leading up to it – the surgery, and the treatments. So, it was nice to be in a small group that had been through that as well, it was healing, a sort of a comradery."
Sub-theme: Personalized support from experts	Participants in the study expressed a strong belief in the therapeutic power of food to manage cancer side effects, especially those related to the GI system. They found the relationship between cancer and diet to be intricate and unique to each individual, posing challenges that were not adequately addressed by mainstream nutritional guidance for	"I found the cooking class is useful. The Dietitian and Chef were good at answering the questions, like, I have had questions about alternative things and they could come up with suggestions for me. They seemed to have a lot of knowledge and be quite enthusiastic about sharing it and made it very accessible at the same time."

	cancer survivors. This lack of clarity led to confusion and, for some, a fear of food. Trust and confidence in nutrition information, delivered in an accessible/easy to understand way, improved when participants received personalized advice from trusted professionals (i.e., Wellness Chef and RD) specializing in diet/nutrition and cancer.<br><br>Several participants emphasized the importance of person-centered approaches to nutrition counseling, particularly to address individualized dietary challenges and GI side effects. The EDIBLE program was seen as filling this gap by providing tailored information and opportunities for personalized advice on coping with GI-related challenges. Participants appreciated the attention given by the Wellness Chef and RD to their personal dietary preferences while still adhering to program guidelines.	"I did feel comfortable reaching out to both of them [Wellness Chef and RD]. I didn't do it as much throughout the course of the program since you are getting the weekly emails [with dietary tips and tricks]. But if I had any questions to help clarify or personalize the recipes, I did email the Chef and he was always very prompt in responding and following up. So, with the two of them they were very welcoming and opening to ask questions… They also helped to problem solve when certain foods didn't work as expected."
Sub-theme: Experiential Learning	In-person cooking demonstrations were the most valued aspect of the EDIBLE program. Participants highlighted the benefits of hands-on learning for better retention of nutritional information and cooking techniques. Engaging in practical activities in a group setting, with the opportunity to ask questions and receive feedback, enhanced their knowledge and skills. This, in turn, increased their confidence and motivation to apply lessons learned in the classroom when cooking at home, and promoted long-term adherence. Indeed, at 3-month follow-up, the majority of participants continued to prepare recipes and utilize culinary skills learned through the EDIBLE program.	"The actual demonstration, the cooking portion, I found the most useful…I retain better by doing it rather than watching or reading it."<br><br>"It was hands on, so you could see what the recipe was going to be, and then you got the chance to make our own little sample of it. So there was a chop board and papillotes and we did a piece of fish, we cut our own vegetables that we wanted, and then he baked it and we discussed our issues at that time and then we ate…that was really good"<br><br>"So, every time we went [to in-person EDIBLE sessions], they did little demonstrations of different foods and recipes for [gastrointestinal] health. Making it together was helpful, so when I came home or whatever, I was confident to make them. I am still actually making them. I am still making the dishes that the Chef made with us. You know, my husband likes them."

Sub-theme: Social support	The group-based environment of the program allowed cancer survivors to interact and learn from each other. Participants found value in this interaction for two main reasons: first, it enhanced the overall educational experience by fostering group-based question and answer sessions and offering opportunities to troubleshoot with others experiencing similar side effects. Second, sharing experiences provided opportunities for cancer survivors to normalize their experiences and validate their struggles with GI issues.	"I think that things like this work so much better in a group. You get a chance to talk about it and get some feedback from the group."<br><br>"Some people may think that they are the only ones who are having this symptom and this is happening to. So, it's always good to talk to other people, and get their input of what there are going through.
**Areas for program improvement**	See sub-themes below	See sub-themes below
Sub-theme: Frequency of in class information and cooking sessions/ demonstrations	The participants universally regarded the in-person sessions as pivotal, highlighting their importance within the EDIBLE program. Consequently, a number of participants expressed a desire for more in-person sessions. However, a small subset of participants also recognized that a higher frequency of in-person sessions might present obstacles or accessibility challenges, especially for individuals who are employed or grappling with considerable side effects induced by treatment, and that for these individuals a different approach to program delivery may be required.	"The people in the group were great, so it was a fun experience, and I think it would have been fun to have more of them. I know that may be a lot to ask, but we had two classes and the Chef sent us recipes in between classes, and he is a great chef. I learned a lot, and I am a pretty good cook, but I found it great to have a professional Chef with us, it was very motivating. So it would have been good to have two or three more classes."<br><br>"At one point I was off sick so I could come and go and stay there as long as I want, but now that I'm working and the class is, the first one I went to it was in the afternoon […] so it's a little bit awkward for me, timing wise. That would be my only reason why I wouldn't attend, because of the timing."<br><br>"I like more the cooking demonstrations, but I'm wondering if it would be possible for the people who are having nasty, nasty side effects of chemo. Maybe you could put it as an option, in case they are able to attend, so partially in-class, partially email, give them the option."
Sub-theme: Tiered program	Participants came into the program with varying levels of nutrition and culinary	"I would say you need to differentiate the comforts of cooking and figure out the

for different cooking comforts/levels	experiences and expertise. Some suggested adopting a multi-tiered approach to the EDIBLE program to reflect such differences. For instance, a few participants in the program possessed robust nutrition knowledge and culinary skills; for these individuals, some expressed a desire for more advanced learning. While reinforcement of their existing dietary practices by experts was validating and helped build confidence in their management, they also wanted opportunities to be challenged and grow through the program. On the other hand, the majority felt at ease with the content and pace of the classes but found themselves intimidated by more knowledgeable peers. A suggested solution to address these varying comfort levels was to implement a tiered approach to the EDIBLE program, allowing individuals to advance through different program levels, progressively building on their knowledge and skills.	levels of people and potentially have different programs. I know this is way too early to know if you are doing that, but with (name of chef), something that I saw, and watched him do it, which is great because you pay attention and he does it very efficiently, is he would meet people at their different levels in one-on-one conversations, but that is a lot to manage."<br><br>"…but when you feel that you are surrounded by cooks which most people are, we feel like we are in high school again. Like you are not doing the right thing. But that is me."
Sub-theme: Personalized approach to sessions/program	The personalized nature of the program was identified as a significant strength, however, this often relied on individual participants expressing a need for personalized supports (either in-class, or via email or telephone). Some felt that a person-centered approach could be systematically integrated into the design of the program itself, with variation from group-to-group, based on that specific groups' needs. Building more one-on-one consults into the larger group-based program was also suggested, opening the opportunity for those reluctant to reach out to the intervention Leads (i.e., Wellness Chef and RD) to express their unique challenges and receive personalized advice (e.g., how to adjust recipes to cook for one). Recognizing that the program was intended to address GI side effects specifically, a minority of participants expressed a need for the program to also consider the vast and intersecting nature of cancer-related side effects – including fatigue, pain, and	"Oh, I know what I would have thought If you're looking for something that I would have changed. I just realized that I would have had an interview of 15-20 minutes with each individual participant while we were down there just to talk about expectations of what they think they're about to receive, what they would like to receive, and then an exit interview of 20 minutes. I know you're doing that now over the phone, but you know in person is always better and I would have done that a little bit closer to the time when we're actually there. So, after the last class I would have asked each person for 15-20 minutes."<br><br>"The only issue with it, and it was personal – as a single person and living alone, the recipes were for more than one person, and I don't eat according to recipes."<br><br>"Maybe more flexibility and adaptability. One think that the program didn't cover

	taste changes – that can affect program adherence.	was what do you do when stuff tastes like metal? You use plastic knives and forks.”

S3. Table H. Qualitative Table for Clinical Outcomes

Theme	Analytic note	Illustrative quotes
Knowledge and confidence	Many participants described improved nutrition knowledge and culinary confidence resulting from their participation in the EDIBLE program. They explained that the program not only imparted practical knowledge but also instilled the confidence to apply that knowledge outside of the EDIBLE program Learning simple yet effective strategies, such as blanching fruits and vegetables for skin and seed removal, showcased an expanded culinary toolkit. Having knowledge and confidence to manage GI side effects improved motivation for long-term adherence, and for some, gave them a newfound sense of control and fostered better self-efficacy.	“Now I have things in my cupboard that I could put together and make something that will not upset my stomach. I know I now eat lighter than I used to, and now I also have the confidence to put things together and make something that will be more satisfying to my body.”<br><br>“Yes, seeing what people do, like watching people cooking on tv, it is harder than it looks. But seeing what the Chef was doing and being able to replicate some of that, not exactly how he was doing, because he is a professional, but I think I did a good job. Also, other things that they were showing, like, the Dietitian was talking about removing the skin when preparing fruits and vegetables, and they were showing really simple ways of doing that. I know it is not a technique, but it is a different way of approaching things. So, things that you can do that will make things better. So, yes, I would say definitely I’m more confident, I learned a

		lot of the recipes and the techniques, so I do think that helps a lot."
Impact to Diet and Dietary Habits	For most participants, the program had a positive influence on their dietary behaviors. Some explained that knowledge gained through the program helped resolve uncertainties about what would/would not aggravate their GI symptoms, reducing overall fear of food and promoting increased food and nutrient intake. Many reported a heightened commitment to healthier eating practices learned in the program and described the deliberate exclusion of certain items from their diets in favor of options deemed less disruptive to their digestive system. However, a minority of participants did not maintain changes to their diet. Typically, this was because they were already adhering to a nutrition regimen akin to that advocated in the program.	"…you can't eat this, and you can't eat that. So, I was a little hesitant about eating [before joining the EDIBLE program]."<br><br>"I think I have been eating healthier. I notice what I eat and all that soluble and insoluble fiber, and whatever gives me gas I tend to stay away from them, you know? So, I am more aware…It makes you feel good, I mean I know what to use and what to do. So, I know that if I eat the tomato or apple with the peel it will make me have gas, so I peel the tomato, I peel the apple. So, I am more aware, it has taught me a lot."<br><br>"One of the main things was skins on fruits and vegetables, I now peel everything, and I didn't do that before. And I also eat fewer berry related fruits. I know they cause a little bit of bloating and so forth. All that insoluble fiber, that's what I try to avoid now. I have paperwork [from EDIBLE] that was given and every so on I review that to see what I should or should not be eating."<br><br>"I also cut back my sugar, especially anything that creates gases, like pop and anything of that kind of stuff, I also eliminated. I also started eating smaller meals and that has been helpful too."<br><br>"Honestly, I would say no [I haven't made any changes to my diet]. I had been eating healthy prior."
Impact to digestive irregularities, bowel symptoms, and other side effects	Participants described a range of experiences pertaining to program impact on GI and other cancer-related side effects. Most explained that the program had a positive influence on symptom management with notable improvements to their digestive health and alleviation of bowel symptoms. Many of these participants also noted improvements in their energy levels and general feelings of health. Others	"I noticed that my bowel movements are… because for me, I would either be constipated, or have diarrhea so I would be bouncing back and forth all the time. I notice that now [after the EDIBLE Program] I have a daily bowel movement and I am less bloated and gassy, and I feel better. So it has helped me a lot."<br><br>"oh no, [I'm] much better now, because I know now what [foods] to avoid, you

	reported more modest changes and one participant reporting no discernible impact on GI symptoms; yet despite marginal or no improvements in GI symptoms, participants expressed gratitude for the program's overall impact, including improvements in dietary practices associated with culinary strategies, tips, and techniques (e.g., low-mess recipes, batch cooking) which were acknowledged as instrumental aids in managing fatigue. This enhanced participant engagement in home cooking and led to improvements in their general health and sense of well-being.<br><br>For those who experienced improvements in GI health, many also described subsequent improvements in quality of life, describing a sense of pre-treatment freedom to do such things as run errands and socialize with family/friends without the fear of experiencing GI issues.	know, I don't eat turnip or broccoli or cauliflower – not that I ever loved those things, but I just stay away from them now because I have instant unbelievable gas from them."<br><br>"I feel more energetic, healthier actually. And the [recipes] are helping with my symptoms."<br><br>"Yes, it was nothing major, but I did notice the little things like taking longer for some of the symptoms to return. They have been small but significant changes"<br><br>"And I find that when I am eating food that is healthy, I have more energy than when I was eating food that is not healthy for me, and I'm definitely eating healthier after this program."<br><br>"No, my symptoms have been the same, just sort of erratic…But I do feel healthier"<br><br>"The symptoms have lessened. I can do more things and I don't have to worry as much when I go out. So, I can be back in the world and that is huge."
